# Efficacy of different psychological interventions for the treatment of inflammatory bowel disease: a systematic review and network meta-analysis

**DOI:** 10.3389/fmed.2025.1630034

**Published:** 2025-10-07

**Authors:** Hao Wang, Jiali Ding, Guangxu Liu, Guangjun Sun, Xiaoyu Zhang, Wenjie Xiao, Yifan Cai, Aizhen Lin

**Affiliations:** ^1^Hubei University of Chinese Medicine, Wuhan, China; ^2^Jingmen People’s Hospital, Jingmen, China; ^3^Renshou County Hospital of Traditional Chinese Medicine, Meishan, China; ^4^Hubei Provincial Hospital of Traditional Chinese Medicine, Affiliated Hospital of Hubei University of Chinese Medicine, Hubei Province Academy of Traditional Chinese Medicine, Wuhan, China; ^5^Hubei Shizhen Laboratory, Wuhan, China

**Keywords:** inflammatory bowel disease, psychotherapy, network meta-analysis, depression, quality of life

## Abstract

**Objective:**

National guidelines for inflammatory bowel disease (IBD) recommend psychotherapy, but the relative efficacy of different psychological interventions is unclear. To address this issue, we conducted a systematic review and network meta-analysis.

**Methods:**

The PubMed, Cochrane Library, Embase, and Web of Science databases were systematically searched for randomized controlled trials (RCTs) from the databases’ inception to October 11, 2024. The primary outcomes were depression, anxiety, and stress levels, and the secondary outcomes were disease activity and quality of life. Two reviewers independently selected studies, extracted data according to pre-specified criteria, and assessed the risk of bias using the Cochrane Collaboration’s risk of bias tool. Network meta-analysis was performed using Stata 16.0 and R. Comparators included usual care (UC), waiting list (WL), and head-to-head comparisons between psychological interventions.

**Results:**

Nineteen RCTs (1,637 participants) evaluating 12 interventions were included. Compared with WL, mindfulness interventions (MI) (SMD −0.63, 95% CI −1.20 to −0.05) and cognitive behavioral therapy (CBT) (SMD −0.54, 95% CI −0.90 to −0.17) reduced depression. Compared with WL, acceptance and commitment therapy with a compassion-focused group component (SMD −1.15, 95% CI −2.21 to −0.05), acceptance and commitment therapy (SMD −1.01, 95% CI −1.83 to −0.16), and CBT (SMD −0.75, 95% CI −1.41 to −0.09) reduced anxiety. For QoL, MI improved outcomes versus WL (SMD 2.21, 95% CI 0.25–4.12) and versus UC (SMD 1.82, 95% CI 0.53–3.10). No significant differences were detected for stress or disease activity versus WL or UC (where available). SUCRA rankings suggested that MI ranked highest for depression and QoL, compassion-focused ACT ranked highest for anxiety and disease activity, and CBT ranked highest for stress.

**Conclusion:**

Psychological interventions appear to provide adjunctive benefits for people with IBD. MI shows consistent advantages for depression and QoL; ACT (with or without a compassion-focused component) and CBT reduce anxiety; CBT ranks favorably for stress. Effects on disease activity remain uncertain, and further high-quality trials are warranted.

**Systematic review registration:**

https://www.crd.york.ac.uk/PROSPERO/view/CRD4202460005.

## 1 Introduction

Inflammatory bowel disease (IBD), including Crohn’s disease (CD) and ulcerative colitis (UC), is a chronic inflammatory disease of the gastrointestinal tract with an increasing prevalence worldwide (1). UC is typically characterized by increased stool frequency accompanied by rectal bleeding, whereas CD may present with abdominal pain, diarrhea, weight loss, fever, or perianal disease, reflecting its heterogeneous clinical manifestations (2). Its pathologic factors are complex, involving intestinal microecological dysregulation, intestinal immune dysfunction, and psychosocial factors (3–5); However, the mechanisms of interaction between multiple factors in the development of the disease are poorly understood. As a result, IBD is difficult to control clinically, and its recurrent episodes have a significant impact on patients’ social functioning and quality of life (QoL) (6), as well as a significant economic burden on the social health care system (7).

Pharmacological and surgical therapies remain the cornerstone of IBD management. Several drug classes have demonstrated efficacy in randomized controlled trials (RCTs), including 5-aminosalicylic acid (5-ASA) agents such as mesalazine (8, 9), glucocorticosteroids such as budesonide (10, 11), immunomodulators such as azathioprine (AZA) (12) and 6-mercaptopurine (6-MP) (13, 14), and advanced therapies [biological agents such as infliximab (15, 16) and vedolizumab (17, 18)]. Meta-analyses confirm that these treatments are effective relative to placebo or comparator therapies; however, no medical therapy can eliminate the risk of relapse entirely, and adverse events are possible with any intervention (19–23). All licensed IBD therapies to date have a significant proportion of non or partial responders. Therefore, optimizing patient quality of life not only requires effective control of intestinal inflammation, but also comprehensive management of psychological comorbidity. While these limitations do not diminish the essential role of pharmacological and surgical strategies in controlling intestinal inflammation, they highlight the need for complementary approaches that address broader aspects of patient well-being.

In this context, the psychological burden of IBD has received increasing attention. Compared with the general population, people with IBD experience higher rates of depression, anxiety, and stress, which are associated with worse QoL, persistent symptom burden (e.g., fatigue and pain even in endoscopic/histological remission), reduced adherence, and greater healthcare use (24, 25) This bidirectional brain–gut relationship—mediated by neuroimmune and neuroendocrine pathways—suggests that psychosocial factors arise as consequences of chronic illness and may also contribute to exacerbations (24, 25). Contemporary guidance therefore supports integrating psychological care within a biopsychosocial model to improve QoL and manage symptom burden (e.g., fatigue, unexplained pain, mental health conditions), alongside standard medical/surgical management (26–28). Observational evidence further indicates that such integration can reduce healthcare utilization and may favorably influence the natural history of disease, although this hypothesis requires confirmation in well-designed trials (25).

A variety of psychological interventions have been studied in IBD, including cognitive behavioral therapy (CBT) and gut-directed hypnotherapy, which primarily improve coping and reduce psychological distress rather than directly modifying inflammatory activity (29, 30). While previous meta-analyses have provided valuable pairwise comparisons for specific therapies (31–33), they cannot determine the relative efficacy across multiple interventions simultaneously. To address this gap, we conducted a network meta-analysis (NMA) to synthesize direct and indirect evidence on the comparative effectiveness of psychological interventions in IBD and to inform prioritization of interventions for clinical practice.

## 2 Methods

The algorithm for the network meta-analysis followed the recommendations of the list of guidelines for reporting systematic evaluations and meta-analyses (PRISMA) (34). The study protocol was registered on Prospero, an international prospective systematic evaluation registry (CRD42024600059).

### 2.1 Search strategy

We searched PubMed, Embase, Cochrane Library, and Web of Science databases, restricting the time frame of the literature to the time of the creation of each database to October 11, 2024, and restricting the language to English. The search was performed with a combination of search terms as subject + free words, using the following medical search terms: Inflammatory Bowel Diseases, Inflammatory Bowel Disease*, Psychotherapy, Psychothera*. The specific search strategy used is described in Supplementary Figure S1.

### 2.2 Inclusion and exclusion criteria

Literature that met the following criteria was included in this study: (1) Study population: patients (≥18 years old) who had a diagnosis of IBD, confirmed by endoscopy, histological examination, and the third edition of the European guidelines for the diagnosis and treatment of UC and CD (35); (2) Interventions (each group using one of the psychological interventions mentioned below): Cognitive Behavioral Therapy (CBT), Acceptance and Commitment Therapy (ACT), Mindfulness Interventions (MI), Relaxation Training, Hypnotherapy (HT), and Mindfulness-Based Stress Reduction (MBSR); Control: Usual Care (including usual care, usual treatments, and unprofessional psychotherapies), Waiting List (WL, delayed access to the intervention while continuing UC); or intercomparisons between different psychological interventions; (3) Type of Study: randomized controlled trials; (4) Outcome indicators and diagnostic criteria: depression, anxiety, stress, disease activity, quality of life. The diagnosis of depression was based on the Beck Depression Inventory, Second Edition (BDI-II), Depression Anxiety Stress Scales-21 Items (DASS-21), Hospital Anxiety and Depression Scale-Depression Subscale (HADS-D), visual analog scale (VAS), and Symptom Checklist-90-Revised (SCL-90-R); and for the diagnosis of anxiety, refer to the Generalized Anxiety Disorder-7 (GAD-7), DASS-21, Hospital Anxiety and Depression Scale-Anxiety Subscale (HADS-A), SCL-90-R, State–Trait Anxiety Inventory (STAI); Diagnosis of stress was made with reference to scales such as DASS-21, Perceived Stress Scale (PSS); diagnosis of disease activity was made with reference to Crohn’s Disease Activity Index (CDAI), Short Crohn’s Disease Activity Index (SCDAI), Harvey-Bradshaw Index (HBI), Patient-Reported Outcome (PRO2), and Mayo Score; for the diagnosis of quality of life, refer to the Inflammatory Bowel Disease Questionnaire (IBDQ), Short Inflammatory Bowel Disease Questionnaire (IBDQ), Short Inflammatory Bowel Disease Questionnaire (SIBDQ), Health-Related Quality of Life (HRQOL), and EuroQol Five Dimensions Five-Level (EQ-5D-5L) scales (Table 1).

**Table 1:** Outcome domains and instruments used across included trials.

Domain	Instruments used in included RCTs	Typical range/format	Direction of scoring*
Depression	BDI-II; HADS-D; DASS-21 (depression); SCL-90-R (depression); VAS	BDI-II 0–63; HADS-D 0–21; DASS-21 subscale 0–21 (some studies report ×2 → 0–42); SCL-90-R subscale (instrument standard); VAS 0–100 mm (or 0–10 cm)	Higher = worse depressive symptoms
Anxiety	GAD-7; HADS-A; STAI (State/Trait); DASS-21 (anxiety); SCL-90-R (anxiety)	GAD-7 0–21; HADS-A 0–21; STAI 20–80; DASS-21 subscale 0–21 (×2 → 0–42 in some studies)	Higher = worse anxiety
Stress	DASS-21 (stress); PSS-10/PSS-14	DASS-21 subscale 0–21 (×2 → 0–42 in some studies); PSS-10 0–40/PSS-14 0–56	Higher = worse perceived stress
Disease activity	CDAI; SCDAI; HBI; Mayo score; Short Mayo; PRO2	CDAI/SCDAI/HBI: higher = more active disease; Mayo 0–12; Short Mayo 0–9; PRO2 (2-item composite, study-specific scaling)	Higher = more active disease (lower = remission)**
Quality of life (QoL)	IBDQ; SIBDQ (Short IBDQ); IBDQ-UK; EQ-5D-5L; study-specific HRQoL	IBDQ 32–224; SIBDQ 10–70; IBDQ-UK (instrument-standard scoring); EQ-5D-5L index typically 0–1 (country tariff dependent)	Higher = better QoL (IBDQ family and EQ-5D)

*Scoring conventions follow instrument manuals; some trials report transformed/normalized scores—analyses use the scale as reported in each trial. **Common remission references (illustrative, not enforced across trials): CDAI <150; Mayo ≤2 with no subscore >1.

We excluded literature with the following conditions: (1) animal or cell experiments, case reports, observational studies, scientific experiment plans, reviews, letters, editorials, conference papers, etc.; (2) literature with missing research data or serious errors; (3) duplicate publications; and (4) full text not found.

Two reviewers, WH and DJL, independently assessed titles and abstracts according to the following criteria and searched relevant full-text articles to screen for conforming literature. Differences of opinion encountered during the literature screening process were resolved through discussion or by seeking advice from the third reviewer, LGX.

### 2.3 Data extraction

Two reviewers, WH and DJL, independently extracted the data information of the final included literature, including the first author, year of publication, country, interventions and controls, duration of the course of treatment, duration of follow-up, basic information of the study subjects (Disease type, Sample size, Sex, Age), and outcome indicators.

### 2.4 Quality assessment

The Cochrane Risk of Bias Assessment Tool (RoB2) (36) was used to assess the included studies in 5 aspects: randomization process, deviation from intended interventions, missing outcome data, measurement of the outcome, and selection of the reported result. For each study, 2 investigators, WH and DJL, independently conducted a quality assessment and made judgments of “low risk,” “high risk,” and “possible risk” for the above 5 aspects. Literature in disagreement was evaluated through discussion or advice from a third researcher, LGX, and the results are presented in a risk of bias graph.

### 2.5 Statistical analysis

All analytical procedures of Network Meta-Analysis were done using Stata 16.0 and R software (ver. 4.4.1). The network Meta-analysis was performed using R software (version 4.4.1) with the gemtc package (version 1.0–1) in conjunction with the JAGS software using Markov Chain Monte Carlo (MCMC) method based on a Bayesian framework (37–39). Four Markov chains were simulated with an initial value of 2.5, a refinement iteration step of 1, 5,000 pre-simulation iterations for annealing, and 20,000 iterations to achieve model convergence, and the Deviation Information Criterion (DIC) was used to compare the model fit and global consistency (if the absolute value of the DIC for consistency and inconsistency was less than 3, then the consistent model was applied) (40); in the presence of a closed-loop mesh, we analyzed local consistency using node splitting (41).

We focused on the primary outcomes of depression, anxiety, and stress; and the secondary outcomes of disease activity and quality of life. For those studies that reported multiple follow-up time points, the data closest to the actual end of the intervention were used to represent the “end of treatment” outcome. This approach ensured that we were able to capture the immediate changes induced by the immediate end of the intervention, without being confounded by other factors that may arise during subsequent long-term follow-up. For continuous data, when the same scale was used, weighted mean differences (WMD) were calculated and 95% confidence intervals (CIs) were reported. If the trial being evaluated used different scales to measure the same outcome, the standardized mean difference (SMD) of the 95% CI was used to synthesize the data. A statistically significant difference was considered to exist if the 95% CI did not include a value of zero. The efficacy of all treatment regimens was analyzed simultaneously using a random-effects model based on a Bayesian framework. The results of the analysis included reticulation plots, cumulative probability rankings, league tables, and “corrected-comparison” funnel plots for each outcome indicator (42). The area under the cumulative ranking curve (SUCRA) was used as an indicator of cumulative ranking probability, and the interventions were ranked according to the size of the SUCRA value, with the closer the value was to 100 percent, the better the intervention was (43).

## 3 Results

### 3.1 Literature search and screening process

**Figure 1:**
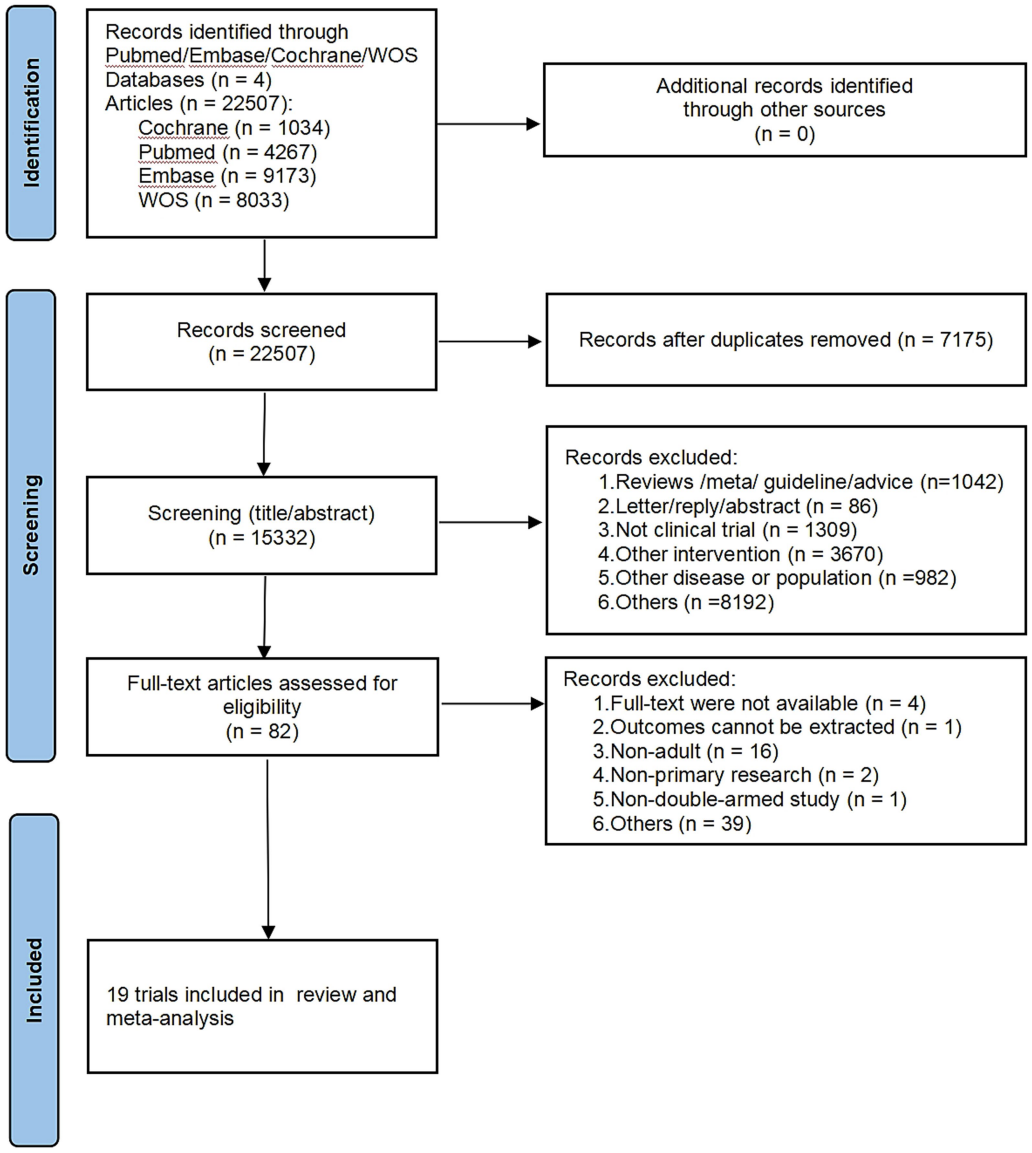
Flow chart of the study identification, screening, eligibility assessment and inclusion processes.

A total of 22,507 documents were retrieved, 7,175 documents were excluded as duplicates, and 15,332 documents were excluded after preliminary reading of titles and abstracts. The remaining documents were read in full text, and were included and excluded strictly according to the inclusion and exclusion criteria, and finally 19 documents were included, and the specific screening process is shown in Figure 1.

### 3.2 Basic characteristics of the included studies

The 19 included studies (30, 44–62) were from nine countries (Australia, Netherlands, Ireland, United Kingdom, New Zealand, China, Israel, United States, and France); a total of 1,637 patients were involved, including 633 males and 1,014 females, with a mean age distribution ranging from 30.1 to 51.9 years old, and the specific interventions included mindfulness-based cognitive therapy (MBCT), ACT, CBT, MI, Multicomponent Cognitive Behavioral Therapy (MulticomponentCBT), Cognitive Behavioral and Mindfulness Intervention (MI + CBT), Relaxation Training, HT, Cognitive Behavioral and Mindfulness Stress Reduction (MBSR+CBT), Acceptance and Commitment Therapy and compassion-based group intervention (MulticomponentACT), Usual Care, Waiting List (WL), Psychoeducation(PE), and Mind–Body Therapy (MBT). 13 studies reported on depression, of which 3 were assessed using the BDI-II scale, 3 using the DASS-21 scale, 5 assessed using the HADS-D scale, 1 assessed using the VAS scale, 1 assessed using the SCL-90-R scale; there were 14 studies reporting anxiety, of which 2 were evaluated using the GAD-7 scale, 4 using the DASS-21 scale, 5 using the HADS-A scale, 2 using the STAI scale, and 1 using the SCL-90-R scale; and there were 7 studies reporting stress, of which 4 used the DASS-21 scale and 3 used the PSS scale; 10 studies reporting disease activity, of which 1 used the SCDAI, 2 used the Mayo score, 1 used the Short Mayo score, 2 used the CDAI, 3 used the HBI, and 2 used the PRO2 scale; and 13 studies reporting quality of life, of which 8 used the IBDQ, 1 used the HRQOL, 1 used the EQ-5D-5L, 2 used the IBDQ-UK, and 1 used the SIBDQ. Of the 19 included RCTs, 7 enrolled patients in remission, 3 enrolled patients with active disease, 7 included mixed populations (both remission and active disease), and 2 did not report baseline disease activity. Information about the basic characteristics of the included literature is presented in Table 2.

**Table 2:** General characteristics of all included studies.

First author	Publication year	Country	Disease type	Baseline disease activity	Sample size	Sex (male/female)	Age (years)	Treatment		Treatment duration	Duration of follow-up	Outcomes
								Intervention	Control			
Bredero et al.	2023	Netherlands	IBD (UC 47%, CD 53%)	Remission	E: 56 C: 57	E: 36/20 C: 34/23	E: 47.3 ± 12.7 C: 46.0 ± 14.8	MBCT, 2.5 h sessions and one 3 h silent session/week	WL	8 weeks	8 weeks	BDI-II, GAD-7, IBDQ
Naude et al.	2024	Australia	IBD	Mixed	E: 61 C: 59	E: 10/51 C: 12/47	E: 34.3 ± 11.3 C: 33.7 ± 10.5	ACT, 8.1 h sessions/week	CBT,8.1 h sessions/week	8 weeks	12 weeks	DASS-21, HRQoL EQ-5D-5L
Wynne et al.	2019	Ireland	IBD (UC 51.9%, CD 48.1%)	Mixed	E: 37 C: 42	E: 17/20 C: 19/23	E: 40.6 ± 11.2 C: 39.9 ± 12.2	ACT, 8 1.5 h sessions/week	Usual care	8 weeks	20 weeks	DASS-21, SCDAI, Short Mayo score
Mikocka-Walus et al.	2015	Australia	IBD	Mixed	E: 90 C: 84	E: 40/50 C: 54/30	E: 46.5 ± 15.7 C: 51.9 ± 16.9	CBT, 2 h sessions/week	Usual care	10 weeks	12 months	HADS-D, HADS-A, CDAI
Artom et al.	2019	U K	IBD	Remission	E: 15 C: 16	E: 5/10 C: 6/10	E: 37.0(31) C: 39.13(33)	CBT, one 1 h and seven 0.5 h individual telephone sessions/8 weeks	Usual care	12 weeks	12 months	GAD-7, HBI, UK IBDQ
Neilson et al.	2016	Australia	IBD	Mixed	E: 33 C: 27	E: 8/25 C: 11/16	E: 38.51 ± 10.62 C: 33.78 ± 12.16	MI, 2.5 h Sessions/8 times and a 7-h weekend session/week	Usual care	8 weeks	32 weeks	HADS-D, HADS-A
McCombie et al.	2015	New Zealand	IBD	Mixed	E: 113 C: 86	E: 38/75 C: 33/53	E: 38.3 ± 12.8 C: 39.6 ± 11.8	CBT, 8 sessions/8 weeks	Usual care	8 weeks	6 months	HADS-D, HADS-A, PSS, HBI, HRQOL
Xi et al.	2022	China	IBD	NR	E: 20 C: 20	E: 14/6 C: 13/7	E: 30.1 ± 16.1 C: 30.8 ± 13.4	MI, 0.5 h training/2 times/d	Usual care	12 weeks	12 weeks	IBDQ
Bernabeu et al.	2021	Spain	IBD	Mixed	E: 60 C: 60	E: 28/32 C: 19/41	E: 44.5 ± 11.81 C: 42 ± 11.65	MulticomponentCBT, 1.5 h sessions/week	Usual care	8 weeks	ND	HADS-D, HADS-A, PSS, CDAI, MAYO SCORE, IBDQ
Regev et al.	2023	ISRAEL	CD	Active	E: 60 C: 60	E: 20/40 C: 25/35	E: 34.4 ± 11.7 C: 33.6 ± 9.7	MI + CBT, 1 h sessions/7 times/week	Usual care	12 weeks	12 weeks	PSS
Evertsz et al.	2017	NETHERLANDS	IBD	NR	E: 59 C: 59	E: 20/39 C: 23/36	E: 39.4 (19.4–76.5) C: 38.7 (20.1–61.8)	CBT, 1 h sessions/8times/week	WL	ND	16 weeks	HADS-D, HADS-A, IBDQ
Mizrahi et al.	2012	Israel	IBD	Active	E: 18 C: 21	E: 9/9 C: 13/8	E: 35.56 ± 13.45 C: 35.57 ± 12.76	Relaxation, 50 min treatment sessions/3 times/5 weeks	WL	5 weeks	NM	VAS, IBDQ
Romano et al.	2024	Australia	IBD	Mixed	E: 26 C: 29	E: 3/23 C: 3/26	E: 32 ± 8.5 C: 34 ± 10.2	ACT, 1 h sessions/week	PE, 1 h sessions/week	8 weeks	8 weeks	DASS-21, PRO2
Keefer et al.	2013	USA	UC	Remission	E: 25 C: 25	E: 11/14 C: 12/13	E: 38.7 ± 11.8 C: 38.8 ± 12.1	HT, 40 min sessions/week	MBT	7 weeks	12 months	IBDQ
Hoekman et al.	2021	Netherlands	IBD	Remission	E: 30 C: 33	E: 7/23 C: 4/29	E: 32.8 ± 13.0 C: 35.7 ± 11.9	HT, 50 min sessions/6 times/12 weeks	Usual care	12 weeks	10 months	SCL90, IBDQ
Schoultz et al.	2015	UK	IBD	Mixed	E: 22 C: 22	E: 6/16 C: 4/18	E: 48.59 ± 12.05 C: 49.68 ± 15.37	MBCT, 2 h sessions/week	WL	8 weeks	6 months	BDI-II, STAI, IBDQ
Jedel et al.	2022	USA	UC	Remission	E: 20 C: 23	E: 11/9 C: 10/13	E: 44.8 ± 13.5 C: 38.7 ± 10.5	MI, 1.5–2 h sessions/week	Usual care	8 weeks	12 months	BDI-II, STAI
Goren et al.	2022	Israel	CD	Active	E: 55 C: 61	E: 17/38 C: 24/37	E: 33.6 ± 13 C: 32.4 ± 11	MBSR+CBT, 1 h video conferences/7 times/12 weeks	WL	12 weeks	12 weeks	SIBDQ
Ferreira et al.	2024	Spain	IBD (UC 45.3%, CD 54.7%)	Remission	E: 24 C: 29	E: 11/13 C: 13/16	ND	MulticomponentACT, 2 h/session; weekly × 9 (total 18 h)	Usual care	9 weeks	12 months	DASS-21, HBI, UK IBDQ

ND, no data; data are expressed as mean (SD), mean or *n* (%). E, experimental group; C, control group; MBCT, mindfulness-based cognitive therapy; WL, waiting list; ACT, acceptance and commitment therapy; CBT, cognitive behavioral therapy; MI, mindfulness intervention; MulticomponentCBT, multicomponent cognitive behavioral therapy; MI+CBT, cognitive behavioral and mindfulness intervention; PE, psychoeducation; HT, hypnotherapy; MBT, mind–body therapy; MBSR+CBT, cognitive behavioral and mindfulness-based stress reduction; MulticomponentACT, acceptance and commitment therapy and compassion-based group intervention; Remission, disease in remission; Active, active disease; Mixed, included both remission and active disease; NR, not reported.

### 3.3 Results of the methodological quality assessment of the included studies

**Figure 2:**
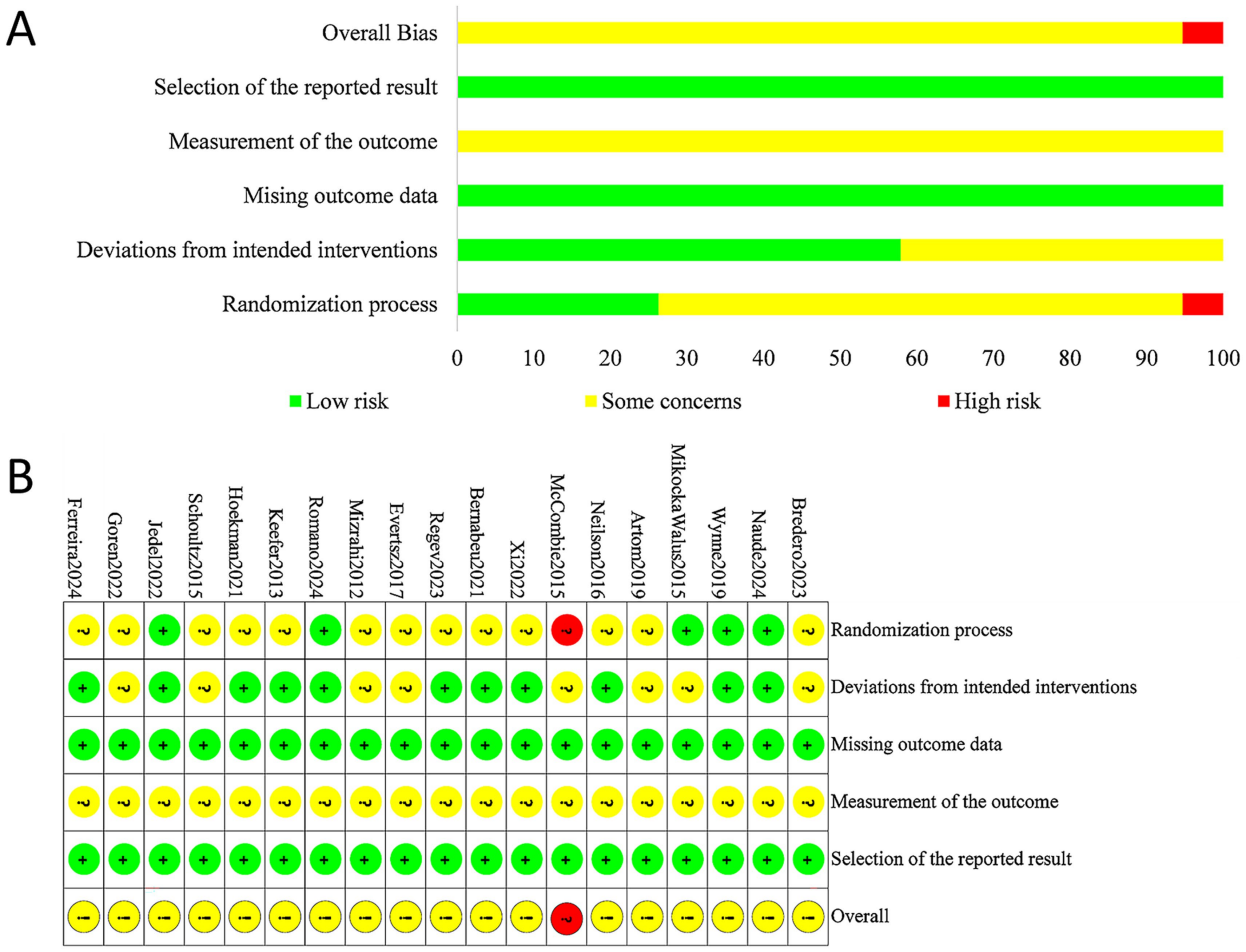
Assessment of risk of bias in the included studies (RCTs). **(A)** Percent of studies with categories for risk of bias; **(B)** summary of the risk of bias in each study.

Based on the Cochrane Collaboration’s Risk of Bias tool, the results of the assessment of the 19 included studies are shown in Figure 2. Regarding the randomization process, 1 study was assessed as high risk due to not implementing allocation concealment, 13 studies were judged as having “some concerns” because they did not report the random allocation method or concealment, and the remaining 5 studies were rated as low risk. For deviations from intended interventions, 8 studies were rated as having “some concerns” due to the absence of blinding or the use of a waiting list as the control group, while 11 studies were at low risk. All studies were at low risk for missing outcome data and outcome measurement. Selective reporting was unclear across all studies and therefore judged as “some concerns.” Taken together, while only one study was formally rated as high risk, the absence of allocation concealment and blinding in the majority of included trials indicates that risk of bias cannot be excluded. It should also be noted that blinding is inherently challenging in psychological intervention studies, as both participants and therapists are usually aware of treatment allocation. Therefore, the findings should be interpreted with caution.

### 3.4 Net analysis results

#### 3.4.1 Network evidence map

**Figure 3:**
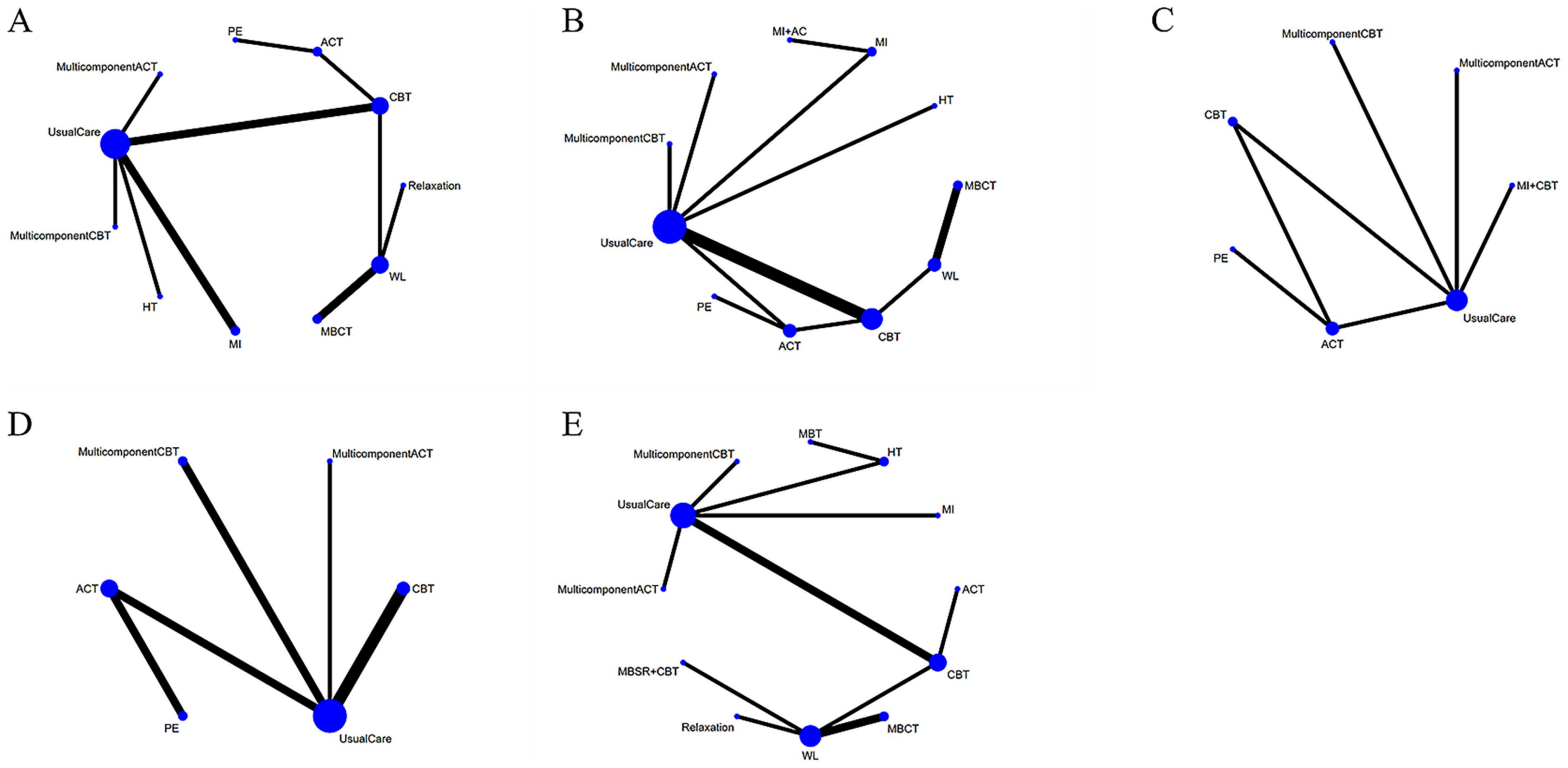
Network plots at the end of treatment. **(A)** Depression; **(B)** anxiety; **(C)** stress; **(D)** disease activity; **(E)** quality of life. The size of the nodes relates to the number of participants in that intervention type. And the thickness of lines between the interventions relates to the number of studies for that comparison.

The 19 included studies covered 12 different psychological interventions: MBCT, ACT, CBT, MI, MulticomponentCBT, MI + CBT, relaxation training, HT, MBSR+CBT, multicomponentACT, PE, and MBT. A network structure diagram comparing the efficacy of the different psychological interventions at the end of treatment is shown in Figure 3. In the figure, the thickness of the lines is proportional to the amount of literature on two-by-two comparisons, and the size of the diameter of the circles is proportional to the number of participants included in the intervention.

#### 3.4.2 Depression

**Figure 4:**
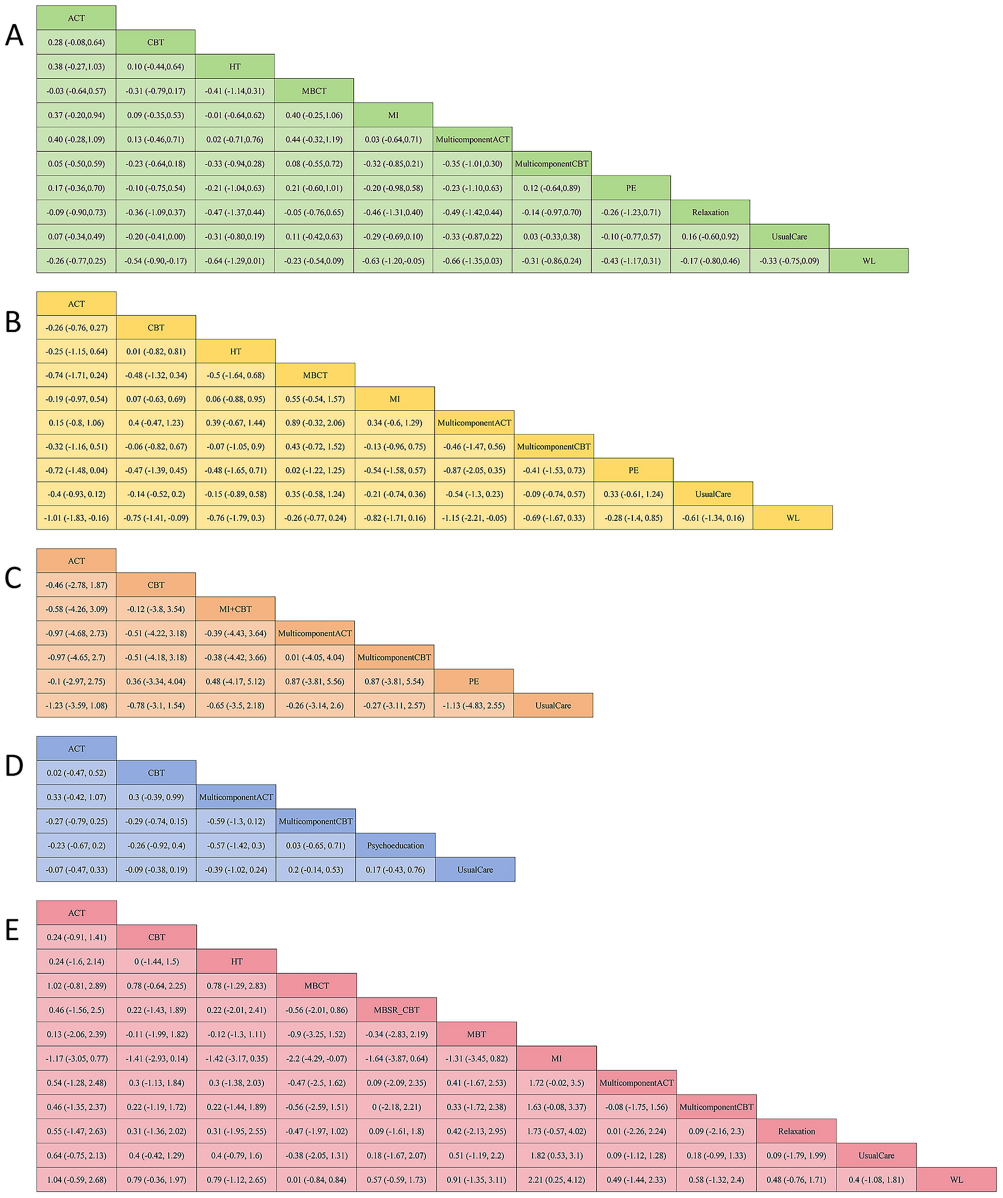
Pooled estimates of the network meta-analysis at the end of treatment. Effect estimates are presented as pooled WMD or RR with 95% CIs. **(A)** Depression; **(B)** anxiety; **(C)** stress; **(D)** disease activity; **(E)** quality of life.

**Figure 5:**
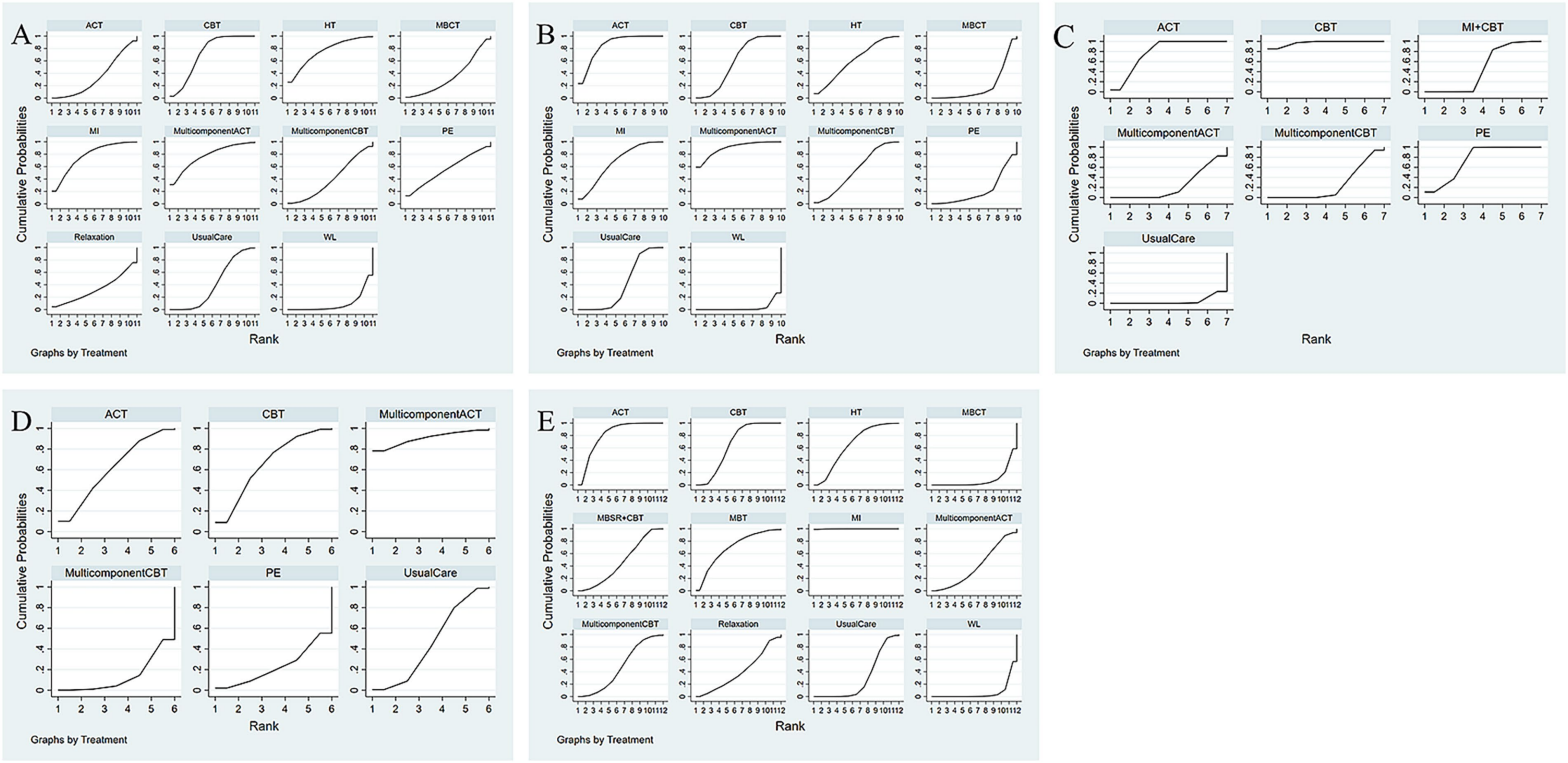
Comparative effectiveness of different interventions surface under the cumulative ranking curves (SUCRA) at the end of treatment. Effect estimates are presented as pooled WMD or RR with 95% CIs. **(A)** Depression; **(B)** anxiety; **(C)** stress; **(D)** disease activity; **(E)** quality of life.

Thirteen studies have reported the effects of nine psychological interventions on depression with a total of 1,201 participants (44–46, 48, 49, 51, 52, 54, 56–61). The network structure diagram between different interventions is shown in Figure 3A. The results showed that MI (SMD = −0.63, 95% CI: −1.20, −0.05) had a significant improvement in depression at the end of the treatment compared to the WL. CBT (SMD = −0.54, 95% CI: −0.90, −0.17) had a significant improvement in depression at the end of treatment compared to the WL. Other two-by-two intervention differences were not statistically significant (Figure 4A). Based on the cumulative probability results, MI (SUCRAs: 77.2%), MulticomponentACT (SUCRAs: 77.0%), and CBT (SUCRAs: 71.7%) may be the three most optimal measures in terms of improving depression (Figure 5A). The comparison-adjusted funnel plot appeared largely symmetrical, suggesting small-study effects are unlikely (Supplementary Figure 1A).

#### 3.4.3 Anxiety

Fourteen studies have reported the effects of nine psychological interventions on anxiety with a total of 1,263 participants (44–52, 54, 56, 58–61). The network structure diagram between different interventions is shown in Figure 3B. The results showed that the MulticomponentACT (SMD = −1.15, 95% CI: −2.21, −0.05) had a significant improvement in anxiety at the end of the treatment compared to the WL. ACT (SMD = −1.01, 95% CI: −1.83, −0.16) significantly improved anxiety at the end of treatment compared to the WL. CBT (SMD = −0.75, 95% CI: −1.41, −0.09) had a significant improvement in anxiety at the end of treatment compared to the WL. Other two-by-two intervention differences were insignificant (Figure 4B). Based on the cumulative probability results, the MulticomponentACT (SUCRAs: 89.9%), ACT (SUCRAs: 85.2%), and MI (SUCRAs: 67.1%) may be the optimal three interventions in terms of improving anxiety (Figure 5B). The comparative-corrected funnel plot presents a largely symmetrical picture, with studies distributed roughly symmetrically on either side of the midline and a small sample effect less likely (Supplementary Figure 1B).

The node-splitting method of analyzing the endings in the presence of closed loops revealed that all *p*’s were >0.05, indicating that there was no local inconsistency.

#### 3.4.4 Stress

Seven studies have reported the effects of six psychological interventions on stress with a total of 737 participants (46–48, 52, 54, 55, 58). The network structure diagram between different interventions is shown in Figure 3C. The results showed no statistically significant difference between two-by-two comparisons between ACT, CBT, MulticomponentCBT, MI + CBT, PE, MulticomponentACT, and Usual Care (Figure 4C). Based on the cumulative probability results, CBT (SUCRAs: 97.2%), ACT (SUCRAs: 78.1%), and PE (SUCRAs: 74.7%) may be the three most optimal measures in terms of improving stress (Figure 5C). The comparison-correction funnel plot presents a largely symmetrical picture, with studies distributed roughly symmetrically on either side of the midline and a small sample effect less likely (Supplementary Figure 1C).

Nodal split analyses were used to confirm consistency between the two intervention programs in any closed loop. Inconsistent estimates also existed for comparisons of Acceptance and Commitment Therapy with CBT (*p* < 0.05), ACT with Usual Care (*p* < 0.05), and CBT with Usual Care (*p* < 0.05).

#### 3.4.5 Disease activity

Ten studies reported the effects of five psychological interventions on disease activity with a total of 935 participants (44, 47, 48, 50, 52, 54, 58). The network structure diagram between different interventions is shown in Figure 3D. The results showed no statistically significant difference between two-by-two comparisons between ACT, CBT, MulticomponentCBT, PE, MulticomponentACT, and Usual Care (Figure 4D). Based on the cumulative probability results, MulticomponentACT (SUCRAs: 90.4%), CBT (SUCRAs: 65.8%), and ACT (SUCRAs: 61.1%) may be the optimal three measures in terms of improving disease activity (Figure 5D). Comparison-correction funnel plots present a largely symmetrical picture, with studies distributed roughly symmetrically on either side of the midline and a small sample effect less likely (Supplementary Figure 1D).

#### 3.4.6 Quality of life

Thirteen studies have reported the effects of nine psychological interventions on Qol with a total of 1,104 participants (30, 45, 46, 48, 50, 52–54, 56, 57, 59, 60, 62). The network structure diagram between different interventions is shown in Figure 3E. The results showed that the MI (SMD = 2.21, 95% CI: 0.25, 4.12) had a significant improvement in quality of life at the end of treatment compared to the WL. MI (SMD = 1.82, 95% CI: 0.53, 3.1) had a significant improvement in quality of life at the end of treatment compared to Usual Care. MBCT (SMD = −2.2, 95% CI: −4.29, −0.07) had a poorer improvement in quality of life at the end of treatment compared to its mindfulness intervention. The two-by-two intervention difference was not statistically significant (Figure 4E). Based on the cumulative probability results, MI (SUCRAs: 99.9%), ACT (SUCRAs: 81.2%), and MBT (SUCRAs: 69.7%) may be the optimal three measures in terms of improving quality of life (Figure 5E). The comparison-correction funnel plot presents a largely symmetrical picture, with studies distributed roughly symmetrically on either side of the midline and a small sample effect less likely (Supplementary Figure 1E).

## 4 Discussion

This study is the first network meta-analysis comparing the effects of different psychological interventions on depression, anxiety, stress, disease activity, and quality of life in patients with IBD. We analyzed data from 19 RCTs covering 12 psychological interventions, providing the most comprehensive comparative evidence. Results of the end-of-treatment analysis showed that MI were the most effective in improving depression and quality of life, MulticomponentACT ranked highest for alleviating anxiety and reducing disease activity, and CBT had the best ranking for relieving stress. Although some pairwise comparisons did not reach statistical significance, the SUCRA ranking patterns indicate clinically meaningful trends that can guide therapy selection in different patient subgroups. However, despite these encouraging findings, current models of care for IBD rarely integrate psychological therapies as a routine component, and access to trained providers remains limited in many healthcare settings (24, 27). Bridging this evidence practice gap will require multidisciplinary collaboration, policy support, and resource allocation to make effective psychological interventions more widely available. These findings offer novel evidence to inform the integration of targeted psychological interventions into comprehensive IBD management.

When interpreting these findings, the methodological quality of the included RCTs should be considered. Most trials were at low risk for outcome measurement and completeness of data, but many had unclear risk in randomization and allocation concealment, and lacked blinding when using waiting list controls. These factors may introduce performance or detection bias, particularly for subjective outcomes. Although funnel plots were largely symmetrical, residual bias cannot be excluded. Future high-quality RCTs with rigorous randomization, concealment, and blinded assessment are warranted.

Previous studies have shown that MI or mindfulness-based psychotherapies are effective in improving depression or other adverse moods in patients with IBD (45, 51, 62–64), and our findings are consistent with these reports. In our NMA, MI demonstrated a significant advantage over Usual Care and Waiting List in reducing depressive symptoms, aligning with a previous systematic review (65), and ranked highest in SUCRA for this outcome. Several factors may explain this result. First, the majority of MI protocols in the included trials incorporated structured PE, mindfulness meditation, and mindfulness activities (61, 66, 67), which may provide both cognitive and behavioral coping strategies to address illness-related distress. These approaches help patients shift attention from automatic, maladaptive thought patterns to nonjudgmental awareness (68), reduce hyperreactivity to somatic discomfort (69), and foster a more accepting attitude toward life (70). It has also been shown that positive thinking intervention can significantly reduce depressive symptoms and improve psychological adjustment and life satisfaction in patients with a variety of chronic diseases (71–73). Second, compared with some other psychotherapies, MI interventions in our dataset tended to have longer session durations and higher adherence rates, factors which could enhance treatment effects. Neuroimaging evidence also suggests that MI can induce beneficial structural and functional brain changes (74–76), potentially improving emotional regulation and stress adaptation. Finally, MI can also improve social interactions in some patients (77), resulting in more understanding and attention (78), and helping individuals to better regulate adverse emotions (79). Therefore, MI can be regarded as a promising psychotherapy to improve the level of depression in IBD patients.

ACT has also been shown to play a positive role in anxiety symptoms (80), and our findings are consistent with this evidence. In the current network meta-analysis, multicomponent ACT reduced anxiety versus WL and ranked highest on SUCRA for this outcome, aligning with a prior systematic review (81) and suggesting that ACT-based approaches may offer particular benefits for anxiety management in IBD. Conceptually, ACT is a third-wave behavioral therapy grounded in functional contextualism (and informed by Relational Frame Theory); rather than positing that an absence of thoughts, memories, or feelings is problematic, ACT holds that psychological suffering is often maintained by experiential avoidance and cognitive fusion with private events. Treatment aims to increase psychological flexibility through six core processes—acceptance, cognitive defusion, present-moment awareness, self-as-context, values clarification, and committed action (82)—which may directly target maladaptive anxiety responses in IBD. In our dataset, the LIFEwithIBD program explicitly integrated compassion-focused elements alongside ACT processes (48), and emerging evidence suggests that cultivating self-compassion can buffer anxiety and distress in IBD and other chronic illnesses (83, 84). Mindfulness-based approaches (e.g., MBCT) are theoretically distinct from ACT but may also enhance attentional control, decentering, and self-compassion, which could complement ACT mechanisms in practice.

CBT has been applied to patients with various chronic intestinal diseases such as IBD (50, 52, 56), and IBS (85) and accompanied by negative emotions such as stress. CBT has been found to significantly improve negative emotions and relieve stress symptoms in these patients (86). In our updated network meta-analysis, however, CBT did not show a statistically significant difference compared with other psychotherapies, Usual Care, or WL for relieving stress symptoms, a finding consistent with a previously published meta-analysis (32). Nevertheless, CBT achieved the highest SUCRA for the stress outcome, indicating a higher probability of ranking among the most effective options across the network despite imprecise pairwise estimates. This apparent discrepancy reflects the fact that SUCRA is rank-based and sensitive to network geometry and comparator mix (e.g., WL vs. UC), whereas NMA contrasts report effect sizes with uncertainty; in a sparse network with small samples, point estimates that are directionally favorable but have wide CIs can yield high ranks without statistical significance. Clinically, CBT may still be considered for IBD patients with prominent stress symptoms, particularly when tailored to individual needs. Mechanistically, CBT targets maladaptive cognitions (e.g., catastrophizing, negative automatic thoughts) and maladaptive behaviors (e.g., avoidance), using cognitive restructuring (identifying and challenging distorted thoughts) (87, 88) and behavioral techniques (e.g., diaphragmatic breathing, progressive muscle relaxation, relaxation training, activity scheduling, and—in some protocols—hypnosis or music-assisted relaxation) (89, 90) to strengthen adaptive coping and reduce perceived stress.

We found MulticomponentACT to be the most effective psychological intervention to improve disease activity in our current network meta-analysis. ACT improves psychological flexibility through different exercises, including metaphors, mindfulness, value clarification, and engaging in actions dedicated to clarifying values (91). The practice of the six core processes of ACT (92) can trigger a new behavioral pattern that commits individuals to what is valuable in their lives (91), which can help patients face and accept the various experiences they have had with openness and to implement and practice the values into specific short-, medium-, and long-term goals, which can help patients with IBD to improve their self-management skills. Some studies have confirmed that the improved self-management ability of IBD patients can strengthen treatment adherence and have a positive effect on disease control (93). One of the ACT-based trials explicitly integrated a compassion-focused group component (LIFEwithIBD), aiming to reduce defensiveness toward emotions and to encourage engaged participation, which may enhance clinical outcomes (48). The other ACT trials targeted psychological flexibility via acceptance, cognitive defusion, present-moment awareness, values, and committed action, without reporting an explicit compassion module (46, 47, 58). However, despite its top SUCRA ranking, MulticomponentACT did not demonstrate statistically significant superiority over other interventions in direct or indirect comparisons. This may be explained by the relatively small number of studies for each intervention and the diversity of psychological approaches included, which can increase heterogeneity and reduce statistical power. Moreover, variability in patient characteristics—such as baseline disease activity, psychological state, lifestyle, and adherence—likely contributed to differences in treatment response (24, 64, 93). These factors should be considered when interpreting the ranking and planning future targeted trials. With respect to disease activity and biomarkers, evidence remains limited and mixed. For example, Wynne et al. (47) reported no between-group differences in subjective or objective disease activity over time with ACT. In Ferreira et al. (48), all participants were in remission at baseline; ACT did not reduce CRP or fecal calprotectin at end of treatment, although an exploratory Crohn’s disease subgroup (n = 14) showed a reduction in Harvey–Bradshaw Index without biomarker change. Taken together, these patterns support the view that psychological interventions may primarily improve stress, coping, and patient-reported indices rather than directly modifying intestinal inflammation; accordingly, the high SUCRA rank for multicomponent ACT on disease activity should be interpreted as ranking under uncertainty, not as proof of superiority.

MI has been recommended in the United States to improve quality of life (94). Several studies have demonstrated the positive impact of MI on quality of life (53, 75). Previous meta-analyses also showed significant improvements in quality of life with MI compared with WL or Usual Care (33, 95). Our updated network meta-analysis confirmed these findings, with MI ranking highest for this outcome in SUCRA values. This effect may be related to MI’s capacity to foster an accepting and tolerant attitude toward life through approaches such as PE, mindfulness meditation, and mindfulness activities, enabling patients to better manage disease-related emotional distress and enhance daily functioning.

## 5 Limitation

To the best of our knowledge, this network meta-analysis of 19 randomized controlled trials assessed for the first time the effects of different psychological interventions on depression, anxiety, stress, disease activity, and quality of life in patients with IBD. This study had several limitations: (1) There were only 19 studies that met the inclusion criteria, involving 12 psychological interventions, and most of them were small-sample studies, resulting in limited statistical validity. (2) Only 7 studies were designed to be blinded, and most had a high risk of bias. (3) Most psychotherapies were compared with WL or Usual Care, and there was insufficient evidence for direct comparisons between different psychotherapies. (4) There is insufficient evidence to compare the long-term efficacy of different psychological interventions. (5) While baseline disease activity was generally comparable between intervention and control arms within trials, it differed across studies. In our dataset, 6 trials enrolled patients in remission, 3 enrolled active disease, 8 included mixed populations, and 2 did not report activity status (see Table 2). This between-study variability may affect indirect comparisons and should be considered when interpreting the results. (6) Several trials used WL controls; because WL comparators can yield larger effect sizes than UC, WL-based contrasts—and by extension SUCRA rankings—may overestimate benefits relative to UC. Although WL and UC were modeled as separate nodes and results are reported against the relevant control, the limited number of head-to-head and UC-controlled trials precluded formal adjustment for comparator type.

## 6 Conclusion

This network meta-analysis suggests that mindfulness-based interventions (MI) show the greatest probability of benefit for depressive symptoms and health-related quality of life; ACT-based approaches appear most promising for anxiety; and CBT shows favorable, albeit imprecise, estimates for perceived stress. Importantly, no psychotherapy demonstrated consistent superiority for disease activity or inflammatory biomarkers; therefore, ranking results (SUCRA) should be interpreted cautiously given sparse head-to-head evidence and heterogeneity in comparators (waiting list vs. usual care). Psychological therapies should be viewed as adjuncts to standard medical/surgical care to address psychological comorbidity and improve quality of life, rather than as substitutes for anti-inflammatory treatment. Future research should include adequately powered, multicenter trials with standardized outcomes (including objective disease measures), longer follow-up, and direct comparisons between active psychological interventions, with blinded outcome assessment where feasible.

## Data Availability

The original contributions presented in the study are included in the article/Supplementary material, further inquiries can be directed to the corresponding author.
