# Supplementary figures and images for: Efficacy of different psychological interventions for the treatment of inflammatory bowel disease: a systematic review and network meta-analysis
Source: Front Med (Lausanne). 2025 Oct 7;12:1630034. doi: 10.3389/fmed.2025.1630034 (PMC12537889; doi:10.3389/fmed.2025.1630034)

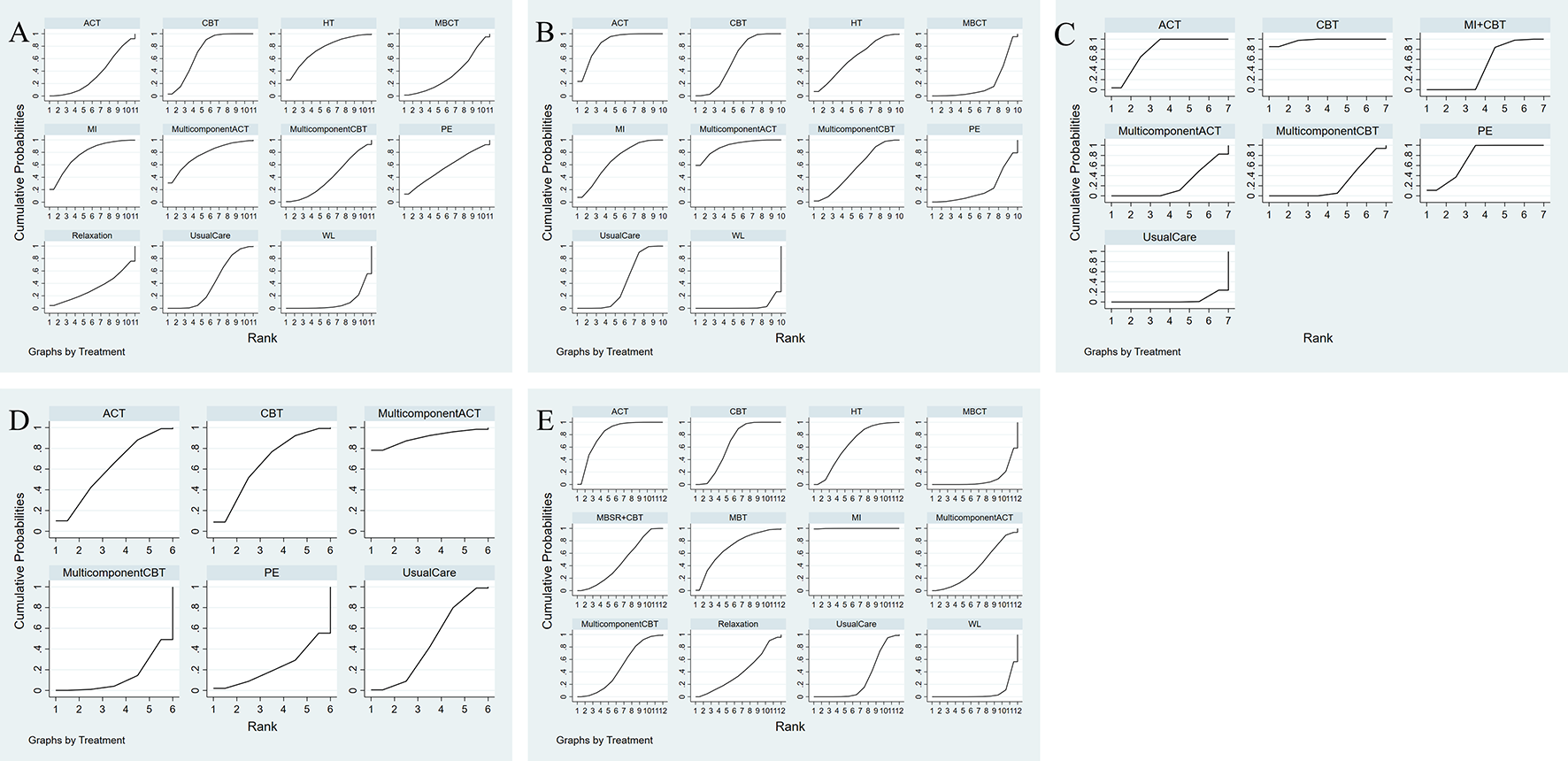

Supplement: Supplementary file 1 [file Image_1.TIF]

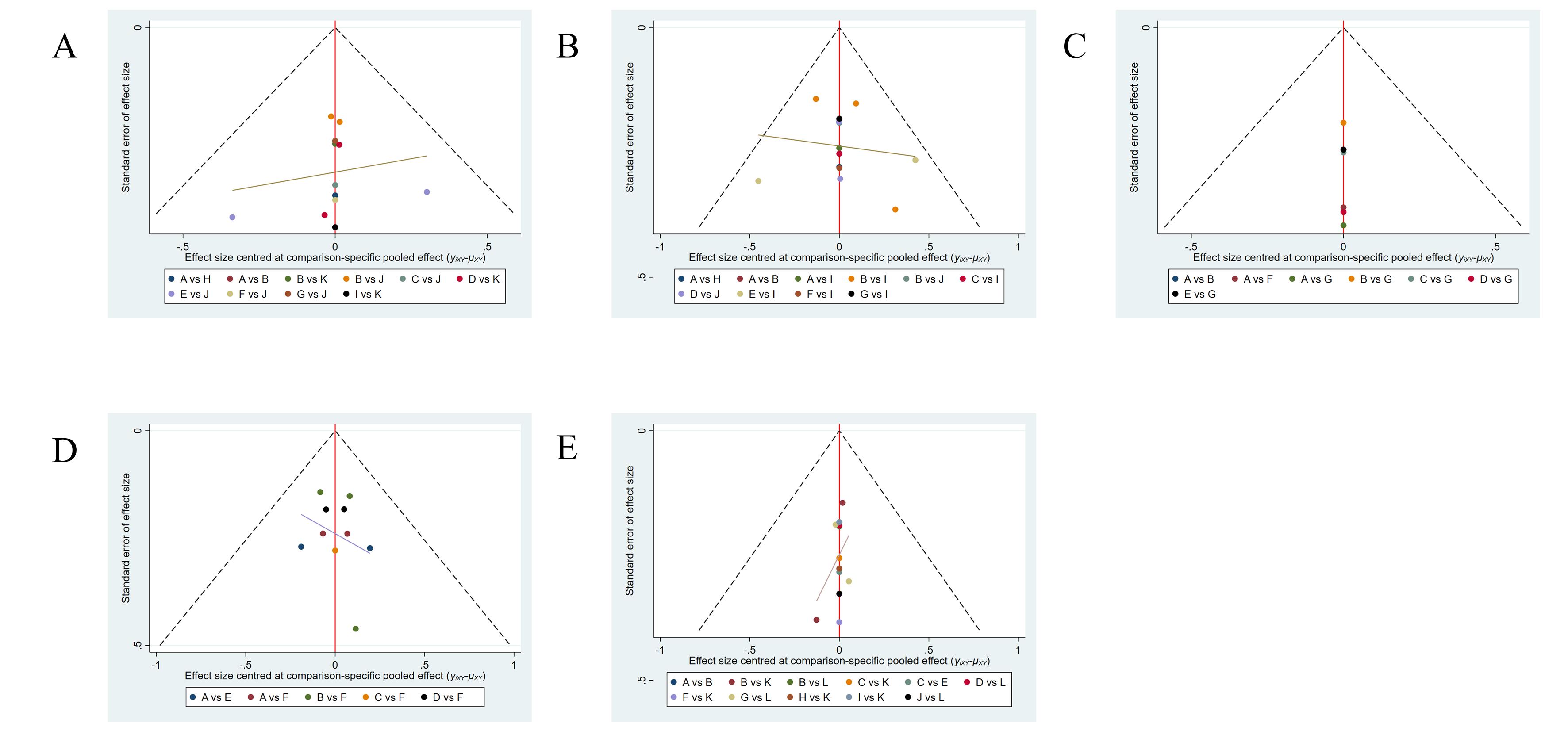

Supplement: Supplementary file 2 [file Image_2.TIF]
